# Protease-Mediated Maturation of HIV: Inhibitors of Protease and the Maturation Process

**DOI:** 10.1155/2012/604261

**Published:** 2012-07-25

**Authors:** Catherine S. Adamson

**Affiliations:** Biomedical Sciences Research Complex, School of Medicine, University of St. Andrews, North Haugh, St. Andrews, Fife KY16 9ST, UK

## Abstract

Protease-mediated maturation of HIV-1 virus particles is essential for virus infectivity. Maturation occurs concomitant with immature virus particle release and is mediated by the viral protease (PR), which sequentially cleaves the Gag and Gag-Pol polyproteins into mature protein domains. Maturation triggers a second assembly event that generates a condensed conical capsid core. The capsid core organizes the viral RNA genome and viral proteins to facilitate viral replication in the next round of infection. The fundamental role of proteolytic maturation in the generation of mature infectious particles has made it an attractive target for therapeutic intervention. Development of small molecules that target the PR active site has been highly successful and nine protease inhibitors (PIs) have been approved for clinical use. This paper provides an overview of their development and clinical use together with a discussion of problems associated with drug resistance. The second-half of the paper discusses a novel class of antiretroviral drug termed maturation inhibitors, which target cleavage sites in Gag not PR itself. The paper focuses on bevirimat (BVM) the first-in-class maturation inhibitor: its mechanism of action and the implications of naturally occurring polymorphisms that confer reduced susceptibility to BVM in phase II clinical trials.

## 1. Introduction

Human Immunodeficiency Virus Type 1 (HIV-1) is the causative agent of the worldwide Acquired Immunodeficiency Syndrome (AIDS) epidemic. Approximately 34 million people were estimated to be living with HIV at the end of 2010. The number of people infected is a consequence of continued large numbers of new HIV-1 infections together with a reduction in AIDS-related deaths due to a significant expansion in access to antiretroviral drug therapy [1]. In the absence of an effective vaccine or cure, antiviral drugs are currently the only treatment option available to HIV-infected patients. Therapeutic regimes commonly termed HAART (highly active antiretroviral therapy) suppress viral replication but do not eradicate the virus; therefore, treatment must be administered on a lifelong basis [2, 3]. HAART consists of the simultaneous use of a combination of three or four different antiretroviral drugs. This combinational approach is required due to the ease with which HIV-1 can acquire drug resistance to a single drug administered as monotherapy [3, 4]. Drug resistance arises due to the high degree of HIV-1 genetic diversity within the virus population (quasi-species) infecting an individual patient. This genetic diversity is created as a consequence of a rapid rate of viral replication combined with the error prone nature of the viral reverse transcriptase (RT), which copies the viral RNA genome into a double-stranded DNA copy and the frequent recombination events that occur during genome replication [3, 5, 6]. HAART is possible due to the successful development and clinical use of more than 20 antiretroviral drugs, which belong to six different mechanistic classes. These drugs primarily target the viral enzymes: RT inhibitors (which fall into two classes based on their mode of action: the nucleoside-analog RT inhibitors (NRTIs) and nonnucleoside-analog RT inhibitors (NNRTIs)), protease (PR) inhibitors (PIs), and an integrase (IN) inhibitor [7–10]. Most clinical treatment regimens use a combination of either a PI or NNRTI with two NRTIs, though since its approval for clinical use in 2007 the first IN inhibitor (insentress) has increasingly been used in therapy regimens. The remaining two mechanistic drug classes each contain one approved drug and target the viral entry process by either blocking viral fusion by targeting the viral gp41 envelope protein or acting as an antagonist against the host cell coreceptor CCR5 [11]. The viral entry inhibitors are in general reserved for salvage therapy. Salvage therapy is required upon treatment failure primarily due to the emergence of drug resistance and to be effective should ideally include at least one new drug targeting a novel site of action. Until a cure for HIV infection is achieved, the continued threat of drug resistance makes the identification and development of a continuous pipeline of new drugs with a novel mechanism of action an ongoing requirement [12]. In this paper we discuss protease-mediated maturation of HIV-1 particles and the strategies to target this step in HIV-1 replication for therapeutic intervention.

## 2. Proteolytic Maturation and Its Role in HIV-1 Replication

**Figure 1:**
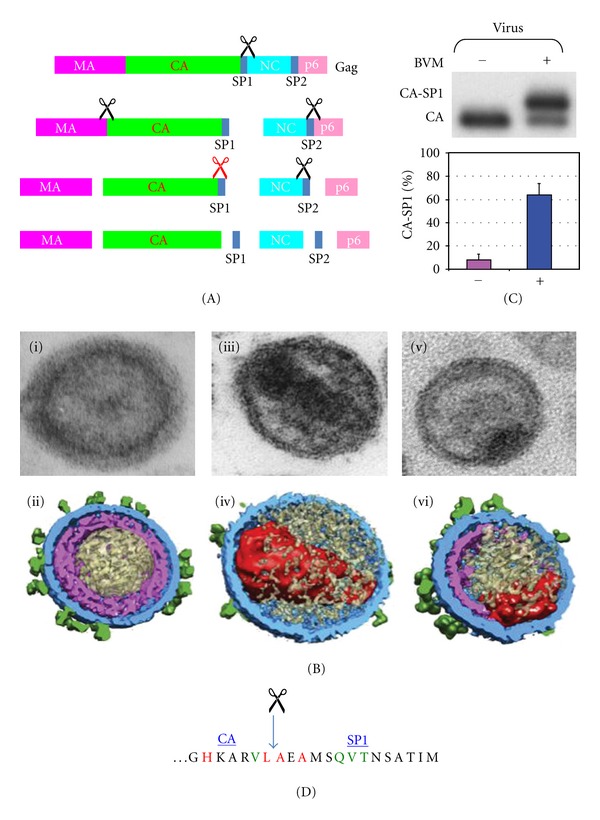
Proteolytic maturation of HIV-1 and its inhibition by bevirimat (BVM). (A) Gag processing cascade, illustrating the order in which the Gag precursor is cleaved by the viral protease. Each cleavage site is indicated by a scissor symbol, the red scissor symbol depicts the cleavage event blocked by BVM. (B) Virion morphology visualized by transmission electron microscopy (i, iii, v) and cryoelectron tomography models generated by segmented surface rendering. The glycoprotein spikes are coloured green, the membrane and MA layer in blue, Gag related shells in magenta, core structures in red, and other internal density in beige (ii, iv, vi). Immature particles (i and ii), mature (iii and iv), and BVM-treated (v and vi). (C) Biochemical data demonstrating accumulation of the uncleaved CA-SP1 precursor in virus particles in the presence of 1 *μ*g/mL BVM. (D) Amino acid sequence at the CA-SP1 junction region; amino acids highlighted in green indicate the highly polymorphic residues to which reduced susceptibility to BVM in clinical trials has been mapped and amino acids highlighted in red indicate those that at which BVM resistance arises *in vitro*. Adapted with permission from Elsevier and the American Society for Microbiology [12, 13].

Proteolytic maturation is essential for the production of infectious HIV-1 virus particles and has been extensively reviewed [16–18]. Particle assembly is driven by the Gag (Pr55^Gag^) polyprotein, which is transported to the cellular plasma membrane where it undergoes higher-order Gag-Gag multimerization. A second polyprotein Gag-Pol (Pr160^Gag-Pol^) is also incorporated into the assembling particle through Gag-Gag interactions. Gag-Pol is expressed via a −1 ribosomal frameshift during approximately 5–10% of Gag translation events. The Pol domain encodes the viral PR, RT, and IN proteins. Gag-Gag multimerization forces membrane curvature and assembly is completed upon budding of the particle from the plasma membrane. Initially, the newly formed particles have a noninfectious immature morphology. However, concomitant with virus budding, PR is activated to facilitate particle maturation. The exact mechanism of PR activation is not clearly understood, but it is known to require Gag-Pol dimerization. Once PR is liberated from the polyprotein through autocatalysis, it cleaves Gag and Gag-Pol into their respective proteins. Cleavage of the Pol domain results in the enzymatic proteins PR, RT, and IN. Cleavage of Gag results in four protein domains: matrix (MA or p17), capsid (CA or p24), nucleocapsid (NC or p7), p6, and two spacer peptides SP1 (p2) and SP2 (p1) (Figure 1(a)). Gag cleavage follows a sequential cascade that is kinetically controlled by the differential rate of processing at each of the five cleavage sites in Gag. The first cleavage creates an N-terminal fragment that contains the MA-CA-SP1 domains and a C-terminal fragment that contains the NC-SP2-p6 domains. Subsequent cleavage events occur at the MA-CA and SP1-p6 sites and finally the CA-SP1 and NC-SP2 sites are cleaved.

 The physical consequence of Gag cleavage is a morphological rearrangement of the non-infectious immature particle to a mature infectious particle containing a conical core, which is generated by a second assembly event upon release of the CA domain (Figure 1(b)). The conical CA core contains the RT and IN enzymes along with the dimeric viral RNA genome in complex with NC and is essential for virus replication upon infection of a new cell. Therefore, correct core formation is essential for the production of infectious particles and this has been shown to be dependent on accurate proteolytic processing of Gag as mutations that disrupt the cleavage of individual sites or alter the order in which sites are cleaved result in aberrant particles that have significantly reduced infectivity. The fundamental role of proteolytic maturation in the generation of infectious particles makes inhibiting this process an attractive target for therapeutic intervention. In this paper we discuss how this has been approached by (i) the successful development and clinical use of PIs which target the PR enzyme itself and (ii) research to develop a novel class of antiretroviral drug termed maturation inhibitors which target the Gag cleavage sites that act as the substrate for PR.

## 3. Protease Inhibitors

### 3.1. Introduction

Protease inhibitors (PIs) target and inhibit the enzymatic activity of the HIV-1 PR. PIs inhibit PR activity to the extent that is sufficient to prevent cleavage events in Gag and Gag-Pol that result in the production of non-infectious virus particles. The development of PIs in the mid 1990s was a critical step forward in the successful treatment of HIV-1 patients. This is because their development provided a second mechanistic class of antiretroviral drug, which made HAART combination therapy possible. PIs have remained a key component of HIV-1 patient treatment regimens right up to the current day. To date, nine PIs have been approved for clinical use, they are saquinavir, ritonavir, indinavir, nelfinavir, fosamprenavir, lopinavir, atazanavir, tipranavir, and darunavir [8] (Table 1).

### 3.2. Protease Inhibitor Design

**Figure 2:**
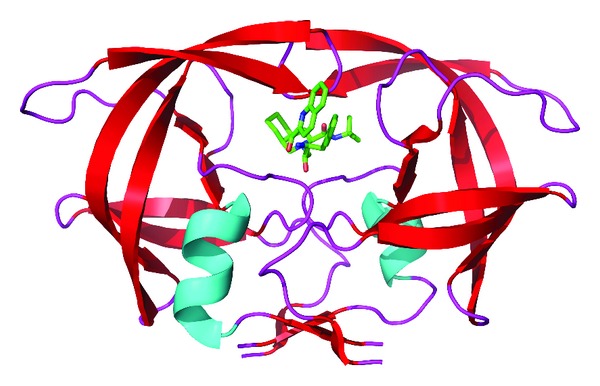
Three-dimensional structure of the HIV protease dimer in complex with the protease inhibitor saquinavir bound at the active site. Adapted by Jerry Alexandrators with permission from Annual Reviews [14].

Design of PIs has been primarily driven by structural knowledge of PR (Figure 2), its substrate, and the chemical reaction of peptide bond cleavage [16]. Like other retroviruses, HIV-1 PR, is related to the cellular aspartyl PR family, which include pepsin and renin. This family of proteases are typified by an active site that uses two apposed catalytic aspartic acid (Asp) residues, each within a conserved Asp-Thr-Gly motif. To function, the cellular PRs form a pseudodimer utilizing two Asp residues from within the same molecule to create an active site. In contrast, retroviral PRs only contain one Asp-Thr-Gly motif and must therefore form a true dimer. Indeed X-ray crystallography has shown that the HIV-1 PR exists as a dimer consisting of two identical monomers [19–21]. The crystal structure of the dimer reveals that four-stranded *β* sheets derived from both ends of each monomer hold the dimer together. A long substrate-binding cleft is created between the monomers and the active site is situated near its centre with the two Asp residues located at its base. Two *β*-hairpin flaps originating from each monomer cover the active site and are thought to function by stabilizing the substrate within the binding cleft.

 The substrate-binding cleft interacts with multiple different substrate cleavage site sequences in Gag and Gag-Pol. The sequence of these sites are at least seven amino acids long and termed P4-P3′, with P1 and P1′ directly flanking the cleavage site [16]. There is no clear consensus amino acid recognition sequence; however, general patterns have been recognised and most substrate sites have a branched amino acid at the P2 site, a hydrophobic residue at P1, and an aromatic or proline at P1′. Instead of amino acid sequence, the topology of the cleavage site is primarily important for their recognition and interaction with PR [22]. Each of the substrate recognition sites has a super-imposable structure, known as the substrate envelope, which fits within the PR substrate-binding cleft. The divergent amino acid sequences of substrate recognition sites do however result in subtle structural differences, which are caused by different side chain protrusions from the substrate envelope. These side chains extend into pockets or subsites in the substrate-binding cleft. Each subsite is named for the corresponding substrate side chain, for example, the S1 subsite corresponds to the P1 side chain. These differences are thought to alter the rate at which cleavage occurs at individual sites in Gag facilitating the regulated proteolytic processing cascade of Gag that is essential for correct particle formation.

 The catalytic mechanism of substrate cleavage requires the Asp residues to coordinate a water molecule that is used to hydrolyze the target peptide (scissile) bond [23]. During the reaction, a transition state intermediate is formed which has been mimicked in the design of most PIs, which are peptidomimetic transition-state analogues. The principle of this design strategy is that the normal peptide linkage [–NH–CO–] is replaced by a hydroxyethylene group [–CH_2_–CH(OH)–], which cannot be cleaved by simple hydrolysis. Saquinavir was the first PI to be approved for clinical use and its design is based on this principle. The following PIs ritonavir, indinavir, nelfinavir, amprenavir, lopinaivr, atazanavir, fosamprenavir, and darunavir also all contain a central core motif of a hydroxyethylene scaffold. The exception is tipranavir, which has a coumarin scaffold and is therefore the only clinically approved PI, which is not a peptidomimetic [8]. Knowledge of the catalytic mechanism and a strategy to generate a transition state analogue was coupled with the ability to cocrystallize candidate inhibitors in complex with PR. This facilitated structure-based drug design that enabled consecutive rounds of lead optimization to develop inhibitors, which competitively bind the active site with affinities to purified PR in the low nanomolar to low picomolar range. The rational design strategy was also used to develop inhibitors that aim to combat problems encountered in the clinic, including poor bioavailability, aberrant side effects, and drug resistance.

### 3.3. Clinical Application and Resistance

Clinical use of PIs began in 1995 with the FDA approval of saquinavir [8]. Saquinavir's approval was closely followed by ritonavir and indinavir in 1996 and nelfinavir in 1997 [8]. *In vitro* studies demonstrated that all of these “first generation” PIs inhibit HIV-1 replication in the nanomolar range in a selection of cell types relevant to HIV-1 infection [24–27]. Initial clinical trials with these drugs were conducted as monotherapy and encouragingly demonstrated declines in HIV-1 RNA levels although the antiviral effect was not sustained for long periods of time due to the rapid acquisition of drug resistance [28–33]. Improved and more sustained reductions in viral RNA levels along with increased CD4 cell counts were obtained when a PI was included in triple therapy combinations with two NRTIs [34–40]. Importantly, these triple-drug regimens (HAART) significantly reduced disease progression and mortality in HIV/AIDS patients [8, 38]. Therefore, the development of PIs facilitated a pivotal step forward in the clinical management of HIV/AIDS and dramatically improved the clinical outcome of the disease.

 Despite the successes, antiviral suppression was not always durable and these early clinical trials highlighted a number of key problems associated with PIs. As indicated above, drug resistance was problematic from the outset and the complex mechanisms of resistance will be discussed in more detail below. Acquisition of drug resistance was compounded by problems with adverse side effects (abnormal lipid and glucose metabolism) and low bioavailability (typical of peptide-like molecules), which led to suboptimal drug concentrations, high pill burdens, and difficulties with patient adherence to treatment regimens [8]. A notable observation to help overcome the pharmacological problems was that ritonavir acts as a potent inhibitor of the cytochrome P450 3A4 metabolic pathway [41]. As a consequence it has been demonstrated that coadministering a low non-therapeutic dose of ritonavir with other PIs leads to dramatically improved bioavailability, half-life, and potency of these PIs [41]. Ritonavir boosting has become a standard procedure when using most PIs in the clinic.

 The next generation of PIs aimed to improve upon the problems highlighted above. The first was amprenavir, which was approved for clinical use in 1999, next came lopinavir which was approved in 2000, followed by atazanavir in 2003, tipranavir in 2005, and lastly darunavir in 2006 [8]. In 2003 amprenavir was subsequently reformulated as the prodrug fosamprenavir, which improved drug plasma concentrations and afforded a lower pill burden [42]. Reduction in pill burden was also achieved by the coformulation of lopinavir with a low dose of ritonavir and further progress in the simplification of drug regimens came with atazanavir, which was the first PI with a once daily dosing regimen. Drug potency has also been improved, in vitro studies have shown atazanavir and darunavir to be particularly potent with IC50 values of between 1 and 5 nM however, tipranavir is the least potent because of its novel nonpeptidomimetic chemical structure [43–47]. Clinical trials demonstrated that the next generation PIs performed well with superior virological efficacy when tested against a placebo or another comparator PI in a background of two NRTIs [8, 48–52]. Finally many of these PIs acquire different drug resistance mutation profiles from the earlier PIs and/or have a higher genetic barrier to resistance.

 Resistance has been encountered for all nine PIs and has been extensively reviewed [8, 53–55]. A current summary of the key mutations acquired by each of them is provided in a data review of HIV-1 drug resistance by the International AIDS Society-USA [15]. The genetic barrier for acquisition of PI resistance is relatively high, that is, it requires two or more amino acid changes to confer significant resistance. This is because PI drug resistance is a stepwise pathway that results in complex interdependent combinations of multiple mutations. All of these interdependent changes are required to act in synergy to confer drug resistance whilst simultaneously maintaining the fitness of the virus.

 The mutations that arise first are referred to as primary or major mutations and they are usually located in the PR substrate-binding cleft or its immediate vicinity. Examples of primary mutations include D30N, G48V, I50L/V, V82A/F/L/S/T, I84V, and L90M [15]. These primary mutations are principally responsible for acquisition of drug resistance by causing conformational changes in and around the active site that prevent inhibitor binding [53]. More specifically, PIs bound to the substrate-binding cleft occupy a similar space as the substrate envelope, but atoms of the PI protrude from this space and interact with residues in PR. Therefore, it has been proposed that drug resistance mutations arise at PR residues involved in these points of contact to inhibit PI binding [56]. In addition to the direct mechanism of resistance described above resistance-conferring mutations may also result in conformational changes to PR beyond the active site and nonactive site mutations can also contribute to drug resistance [53]. Recently rare amino acid insertions, particularly between residues 32 and 42 have been observed to occur more frequently and in correlation with the introduction of atazanavir, lopinavir, amprenavir, and tipranavir into the clinic. The insertions have been proposed to be associated with PI resistance by imposing minor structural changes to the PR flap and substrate-binding cleft, although they always appear in combination with other well-described PI resistance mutations [57].

 Primary mutations are accompanied by secondary or minor mutations, which can be preexisting polymorphisms or acquired after primary mutations. The function of many of these secondary mutations is often not to confer drug resistance *per se* but instead to compensate for the effect of primary mutations, which reduce protease catalytic efficiency and virus replication capacity or fitness [58–62]. Despite their function being less drug specific in action, they are however critical for development of high-level resistance. The secondary mutations are generally located at residues distal from the active site and occur at more than 20 residues of PR [15]. Unlike the primary mutations, which generally occur at highly conserved residues, the secondary mutations, are often polymorphic in PI treatment-naïve patient isolates, a well-documented example is the L63P substitution [58], and thus favour the selection of primary mutations in the presence of drug. Despite the presence of multiple secondary mutations almost all clinical strains of HIV-1 with high-level PI drug resistance display some degree of fitness loss [58, 61, 63, 64].

 Resistance to PIs is a compromise between resistance and PR enzyme function. The mutations in PR described above primarily have an impact on inhibitor binding while still allowing the enzyme to recognise and cleave its Gag and Gag-Pol substrates to some degree. In addition to the changes in PR itself, amino acid changes in the Gag substrate have also been described [54]. These mutations are primarily located at or near to Gag cleavage sites and more specifically the sites in the NC-SP2-p6 region of Gag. Key mutations observed at the NC-SP2 cleavage site are A431V and I437V, which are commonly found in association with the PR primary mutation V82A and key mutations observed at the SP2-p6 cleavage site are L449F and P453L, which are commonly found in association with the PR primary mutations I50V and I84V [65–72]. *In vitro* selection experiments have also shown that mutations at the NC-SP2 cleavage site (A431V, K436E, and/or I437V/T) can also be selected in the presence of PIs without any accompanying resistance mutations in PR [73]. Mutations in Gag located at positions distal to cleavage sites have also been documented [74–76]. The impact of mutations in Gag has been attributed to (i) acting as compensatory mutations that improve fitness defects imposed by PI resistance-conferring mutations in PR and (ii) directly contributing to PI resistance [65–67, 73, 77]. The mechanism by which Gag cleavage-site mutations compensate for a loss in viral fitness is by improving the interaction between the substrate and the mutant enzyme and hence increasing the ability of the mutant PR to cleave [78]. Noncleavage site mutations are thought to improve fitness by causing more broad conformational changes in Gag making cleavage sites more accessible to PR [74–76]. The mechanism by which Gag cleavage-site mutations directly contribute to PI resistance is however not clearly understood [54].

 Despite the complex interdependent combinations of multiple mutations in both PR and its Gag substrate that are required to attain high-level PI drug resistance, many of the PIs have a distinctive primary mutation that can be considered a signatory of drug resistance to that particular PI. For example the D30N mutation is a signatory of nelfinavir resistance, I50L is a signatory of atazanavir resistance, the I50V mutation is a signatory of amprenavir and darunavir resistance, and the G48V mutation is a signatory of saquinavir resistance [15]. Unfortunately, however, many mutations confer drug resistance to multiple PIs leading to broad cross-resistance amongst most PIs [15]. For example, the I84V mutation is the most important as it affects all eight PIs in clinical use and acts as a key mutation for five of them (atazanavir, darunavir, fosamprenavir, indinavir, and tipranavir). Mutations at residue 82 affect all of the PIs except darunavir. The I54V substitution acts as a key mutation for darunavir but it also affects all the other PIs with the exception of nelfinavir and the L90M mutation affects PIs with the exception of darunavir and tipranavir. Cross-resistance is likely due to the fact that although chemically different, most of the PIs were designed using the same basic principle and have similar structures and interactions with the PR substrate-binding cleft. Extensive cross-resistance has serious clinical consequences that threatens the usefulness of PIs and drives an ongoing need for new PIs with improved resistance profiles.

### 3.4. Conclusion

The introduction of PIs into the clinic more than 15 years ago heralded the era of HAART and resulted in a significant reduction in morbidity and mortality among HIV-infected patients. Due to their clinical potency, PIs are still commonly used in treatment regimens, although only three (lopinavir, atazanavir and darunavir) of the nine approved PIs are in widespread use. Despite the clinical benefits, the usefulness of first generation PIs was particularly hampered by toxic side effects and low bioavailability, which resulted in high pill burdens and low patient adherence. A significant advance in resolving these issues was the introduction of low-dose ritonavir boosting, which increases plasma PI levels by inhibiting the cytochrome P450 metabolic pathway. Ritonavir-boosting is itself; however, associated with toxicity; therefore, alternative boosting compounds with improved properties are being developed.

 PI drug resistance is a major cause of therapy failure despite the relatively high genetic barrier to resistance. Unfortunately, PR has proven to be a highly flexible and adaptable drug target due to diverse mutational profiles and the complex interplay between PR and its Gag substrate. Extensive cross-resistance to PIs has also been a key problem that has limited the overall usefulness of the drug class despite the development of new inhibitors such as darunavir with favourable resistance profiles. Therefore, there is a need to develop further novel inhibitors with improved resistance profiles to address these ongoing issues [79]. One strategy to develop such new PIs is to build on the design of existing inhibitors that target the PR active site by introducing novel modifications to established PI chemical entities. One such example is the novel inhibitor GS-8374, which is a modification of a darunavir-like analogue [80]. GS-8374 has been shown to be highly potent with a resistance profile superior to all clinically approved PIs including the parent molecule darunavir [80]. A second strategy is to identify molecules with novel chemical scaffolds, for example PPL-100 is a nonpeptidomimetic inhibitor that incorporates a new lysine-based scaffold and binds the flap region of PR via a novel mechanism [81]. PPL-100 has been shown to have a favourable resistance profile against known PI resistant HIV-1 isolates and its *in vitro* selection pattern results in two previously undocumented mutations T80I and P81S together with two previously reported compensatory mutations K45R and M46I [81, 82]. Allosteric inhibitors that bind a site other than the PR active site via a noncompetitive mechanism of action have also been identified and shown to be effective against both wildtype and PI resistant purified PR [83]. A further novel strategy, discussed below, is to design inhibitors that prevent proteolytic maturation by targeting the Gag substrate rather than the PR enzyme itself.

## 4. Maturation Inhibitors

### 4.1. Introduction

PIs directly target the PR enzyme; however, an alternative approach to inhibiting HIV-1 proteolytic maturation is to identify small molecules that bind its Gag substrate and specifically block individual cleavage events. Such a strategy would be successful because accurate proteolytic processing of Gag is essential for the production of infectious particles as mutations that disrupt the cleavage of individual sites or alter the order in which sites are cleaved result in aberrant particles that have significantly reduced infectivity. Molecules with this mechanism of action have been termed maturation inhibitors and the first-in-class is 3-O-(3′,3′-dimethylsuccinyl)betulinic acid (DSB), also known as PA-457, MPC-4326, or bevirimat (BVM).

### 4.2. Mechanism of Action

BVM specifically inhibits CA-SP1 cleavage, which occurs late in the Gag proteolytic cleavage cascade [84, 85]. This has been demonstrated by a number of key observations: (i) biochemical studies have demonstrated an accumulation of the uncleaved CA-SP1 intermediate in both cell and virus-associated protein fractions from HIV-1 expressing cells treated with BVM [84–86] (Figure 1(c)); (ii) viruses such as HIV-2 and SIV which have a divergent sequence at the CA-SP1 junction are not sensitive to BVM [87]; (iii) the majority of BVM drug-resistance conferring mutations map to the CA-SP1 junction or within SP1 [84–86, 88–95]. A second molecule PF-46396 has been identified that also inhibits CA-SP1 cleavage [96]. Interestingly, although PF-46396 has a similar mechanism of action as BVM, it belongs to a distinct chemical class as it is a pyridone-based compound not a betulinic acid derivative like BVM [96].

 The consequence of BVM blocking SP1 cleavage from the C-terminus of CA is the formation of noninfectious particles with an aberrant morphology [84] (Figure 1(b)). Three-dimensional (3D) imaging of BVM-treated particles by cryoelectron tomography showed that they contain an incomplete protein shell, which has a hexagonal honeycomb lattice in the CA layer that is similar in structure to the Gag lattice of immature virus particles [13]. This partial shell is consistent with the aberrant electron dense crescent inside the viral membrane observed in BVM-treated particles by conventional thin-sectioning electron microscopy [84]. Both imaging techniques also showed most BVM-treated particles to contain an acentric mass, which represents an abnormal core-like structure [13, 84]. The general morphological features of BVM-treated particles are shared by particles generated by the CA5 mutant, which has two amino acid substitutions that completely block CA-SP1 cleavage [84, 97]. However, these particles have a thinner CA layer with no visible evidence of honeycomb lattice organization [13]. The presence of structural organization in the BVM-treated but not the CA5 CA layer suggests that BVM binding stabilizes the immature lattice as well as blocking CA-SP1 cleavage and that both modes of action may potentially contribute to the generation of non-infectious particles [13].

 The assembly state of Gag is a determinant of BVMs activity. BVM does not inhibit CA-SP1 processing in the context of monomeric Gag in solution [84], but instead requires Gag assembly for its activity [84, 98, 99]. Therefore, it can be hypothesized that BVM binds to a pocket formed during Gag-Gag multimerization. Conversely, Gag processing disrupts the putative binding site because BVM has been shown to bind immature but not mature HIV-1 particles [99]. The BVM binding site has been mapped to the CA-SP1 junction within immature virus particles using photoaffinity BVM analogues and mass spectroscopy [100]. This provides the first direct evidence that the BVM binding site spans the CA-SP1 junction and is consistent with previous biochemical and genetic data that have implicated this region of Gag in BVM binding. Indeed, BVM binding is disrupted in a selection of BVM-drug resistant mutations with amino acid substitutions that map to the CA-SP1 junction [100, 101]. Positioning of BVM across the CA-SP1 junction supports a mechanism of action whereby binding blocks access of the viral PR to the CA-SP1 cleavage site. A second related hypothesis is that BVM binding alters the conformation, exposure, or flexibility of this region such that PR cleaves it less efficiently. The binding study [100] also identified a second BVM binding site in the major homology region (MHR) of CA, a region of Gag known to function in virus assembly [17]. The significance of a potential second BVM binding site has yet to be established but may provide an explanation for the observation that at high concentrations BVM inhibits virus particle assembly [102].

 The structure of the BVM binding site remains unknown because this region of Gag has been disordered in X-ray crystallographic studies [103, 104]. The disorder has been attributed to a structural flexibility, which permits higher-order Gag-Gag multimerization during virus particle assembly [105–110]. It is, however, generally accepted that the CA-SP1 region of Gag adopts a *α*-helical conformation. The evidence for a helical structure is based on (i) secondary structure computer modelling predictions [111], (ii) genetic data demonstrating that mutation of key residues predicted to be helix breakers results in a disruption of virus particle assembly [106, 111] and (iii) biophysical and NMR techniques that have shown the CA-SP1 region to have a propensity to adopt a helical conformation under certain environmental conditions [110, 112, 113]. Although the interactions formed by the proposed CA-SP1 junction helices in the Gag lattice are not known, a cryoelectron tomography study of immature particles led to the hypothesis that the CA-SP1 region exists as a six-helix bundle that lies directly below the hexagonal honeycomb CA lattice [114]. Because BVM activity is known to require higher-order Gag-Gag multimerization, it has been suggested that the BVM binding pocket might involve more than one helix and hence bound BVM may occupy a cleft formed between helices [100]. The considerable technical challenges of obtaining high-resolution structural information of the CA-SP1 junction in the context of higher-order multimerized Gag make the prospect of rational drug design using inhibitor cocomplexes not currently possible. However, further understanding of the interactions involved is important for the development of second-generation maturation inhibitors. Such new molecules are now required as clinical development of BVM was suspended in 2010 due to problems with intrinsic BVM drug resistance in HIV-1 infected patients during phase II clinical trials.

### 4.3. Clinical Development and Resistance

BVM was considered an attractive candidate for clinical development because of its potent in vitro activity with a mean IC50 value of 10 nM and its novel mechanism of action, which makes it equally effective against viruses that have acquired resistance to key antiretroviral drugs in clinical use [84]. Additional attributes including promising pharmacological and safety studies in animal models and phase I clinical trials [115] led to the testing of BVM in HIV-1 infected patients. Initial success in these phase II clinical trials demonstrated significant BVM dose-dependent viral load reductions [115]. However, further studies quickly showed that approximately 50% of BVM-treated patients did not effectively respond to the drug and exhibited viral load reductions of less than 0.5 log [93]. Failure to respond was not due to suboptimal BVM plasma concentrations but has been attributed to virological parameters instead.

 Examination of patient-derived virus revealed amino acid assignment at SP1 residues 6, 7, and 8 (Gag positions 369, 370, and 371) is associated with response to BVM [92, 93, 95] (Figure 1(d)). This trio of residues map to the C-terminal half of SP1, which is relatively nonconserved but commonly encodes a QVT (glutamine-valine-threonine) motif in clade B HIV-1 isolates [95]. Patients most likely to respond to BVM are infected with virus encoding the QVT motif, while patients infected with virus encoding polymorphisms at SP1 residues 6–8 are less likely to respond [93]. Studies to investigate the contribution of individual substitutions at SP1 residues 6–8 have shown that mutations at SP1 residue 7 and 8 (e.g., SP1-V7A, -V7M, -T8Δ, -T8N) all confer varying degrees of reduced susceptibility to BVM [88, 92, 94, 95]. Most notably, a critical role for BVM resistance has been attributed to the SP1-V7A polymorphism as it confers full resistance to BVM [88, 92, 94, 95]. BVM susceptibility was not however reduced by mutations at SP1 residue 6 (e.g., SP1-Q6H, Q6A) or the SP1-T8A polymorphism [88, 92, 94, 95]. Therefore, any contribution of these substitutions to reduced BVM susceptibility maybe dependent on the synergistic effects of a combinations of different polymorphisms, i.e. the context of the wider Gag background. Indeed, one study identified five patient-derived virus samples with significantly reduced BVM susceptibility *in vitro* but still encoded the QVT motif [92]. In two of these isolates, BVM resistance has been demonstrated to be conferred by a polymorphism in CA (CA-V230I) situated at the P2 position of the CA-SP1 cleavage site [92] (Figure 1(d)). In the other three isolates, the determinants of reduced BVM susceptibility were not resolved [92], indicating that in some instances the factors conferring BVM susceptibility are likely to be more complex than the parameters that have been established to date.

 The CA-V230I and SP1-V7A substitutions have also been acquired in in vitro BVM drug-resistance selection experiments [88, 90, 91]. In vitro studies have also identified a panel of other BVM-resistance mutations (CA-H226Y, CA-L231M, CA-L231F, SP1-A1V, SP1-A3V, and SP1-A3T) [84–86, 90] (Figure 1(d)). Unlike, the clinically important innate polymorphisms discussed above, these *in vitro* selected BVM-resistance mutations map to residues in the vicinity of the CA-SP1 cleavage site that are highly conserved throughout HIV-1 isolates [86]. As a likely consequence, these mutations have not been observed in most patient-derived virus samples either with [93, 95] or without BVM treatment [92, 95]. However, it should be noted that the most frequently acquired mutation SP1-A1V has been shown not to impose a significant defect on virus replication *in vitro* [86, 89, 90] and replicates efficiently in SCID-hu Thy/Liv mice [116]. Therefore, it remains a hypothetical possibility that the SP1-A1V mutation could be acquired over time in patients that initially respond well to BVM treatment.

 Initial failure to select the key BVM-resistance conferring polymorphisms *in vitro* has been attributed to the experimental conditions utilized [91]; however, later experiments did result in selection of some of the key polymorphic mutations albeit at low frequency [88, 90]. Nevertheless, a recent study used a more sophisticated *in vitro* method of serial passage of quasi-species containing recombinant HIV-1 and deep sequencing that more accurately mimicked *in vitro* the selection of BVM-resistance observed *in vivo* [91]. In hindsight use of this *in vitro* selection method or more extensive testing of the spectrum of activity across a diverse panel of clinical isolates may have more accurately predicted the clinical response to BVM and either led to discontinuation of BVM development at an earlier stage thereby avoiding costly clinical studies or alternatively steered BVM's clinical development to include a genotyping test to screen for preexisting key polymorphisms to enable prior identification of patients most likely to effectively respond to BVM treatment [91].

 The clinically important polymorphisms preexist in the HIV-1 population without prior BVM treatment. This intrinsic resistance has caused problems for BVM's clinical development, which was consequently discontinued in 2010. Genotypic analysis has demonstrated a high prevalence of polymorphisms at the QVT motif and their frequency is dependent on the genetic clade of HIV-1 [94, 95, 117]. In clade B viruses, which are predominant in the US and Europe, polymorphism frequency at the QVT motif has been reported to occur at a rate of ~30–60% [91, 95, 117]. This genotypic analysis matches BVM susceptibility rates in the *in vitro* phenotypic and clinical trial studies discussed above [92, 93, 95]. In nonclade B viruses, QVT polymorphism rates are much higher with rates of >90% [95, 117]. Typically polymorphisms occur most frequently at SP1 residue 7, followed by residue 8, and then residue 6 [91, 94, 95]. The critical SP1-V7A polymorphism has been shown to be largely predominant and occurs at a frequency of ~16% in clade B viruses and ~65–70% in clade C viruses, which are mostly found in Southern Africa [94, 95]. The high frequency of the SP1-V7A polymorphism combined with its known capacity to confer full resistance to BVM therefore poses the biggest threat to the potential effectiveness and clinical development of BVM.

 The prevalence of the key polymorphisms in relation to HAART and the presence of PI resistance mutations has been investigated due to the complex interplay between PR and its Gag substrate. Being a new class of antiretroviral drug BVM was most likely in the first instance to be used as salvage therapy for patients harbouring multidrug resistant HIV-1 isolates. Studies have shown no association between the prevalence of key QVT polymorphisms and HAART treatment experience but in the absence of BVM [91, 92, 117]. One study also reported no association between prevalence of QVT polymorphisms and PI resistance-conferring mutations [92]; however, two other studies with bigger sample sizes demonstrated a higher frequency of BVM resistance mutations in PI resistant patient isolates [117, 118]. The effect of PI resistance on acquisition of BVM resistance *in vitro* has also been investigated [89, 90]. These two studies made different conclusions about the impact of the PI mutations on the temporal acquisition of BVM-resistance conferring mutations, with one study reporting a delay in the emergence of BVM-resistance [89]. The reported differences may be dependent on the type of PI mutations or the study systems used. Interestingly, the other study [90] demonstrated that the PR background influenced the type and diversity of BVM resistance conferring mutations. Viruses with a wildtype PR predominantly acquired the SP1-A1V mutation, whereas viruses with a PI resistance PR acquired a significantly higher prevalence of mutations at the QVT motif (SP1-V7A, V7N, and SP1-T8N), at the polymorphic CA-230 residue (CA-V230I) and also a previously unreported mutation SP1-S5N. The PR genetic background was also found to effect BVM susceptibility and virus replication capacity [90]. While these studies have not fully resolved the complex interplay between PR, the Gag substrate and susceptibility to BVM they clearly demonstrate that this parameter should be considered in future development of maturation inhibitors.

### 4.4. Conclusion

Maturation inhibitors are a novel mechanistic class of antiretroviral drug that target PR cleavage sites in Gag. BVM is the first-in-class maturation inhibitor, which specifically inhibits cleavage of SP1 from the C-terminus of CA. A number of other small molecules that target Gag have also been identified. PF-46396 is a second maturation inhibitor, which also inhibits CA-SP1 cleavage but is chemically distinct from BVM. There are also a small number of molecules that target CA and inhibit assembly of the immature particle and/or the CA core [12]. BVM is however the only molecule that targets Gag, which has been tested in clinical trials. BVM was considered a good candidate for clinical development because of its *in vitro *potency, novel mechanism of action, and good safety profile in animal models and phase I clinical trials. Although initial results of BVM efficacy in HIV-1 infected patients were encouraging, it was quickly established that approximately 50% of patients do not effectively respond to the drug. Failure to respond is due to virological parameters, more specifically, intrinsic polymorphisms primarily located at SP1 residues 6, 7, and 8. These polymorphisms have a high prevalence, particularly in non-clade B HIV-1 isolates. The existence of BVM-resistance conferring polymorphisms in BVM-treatment naïve patients severely limits the clinical usefulness of BVM and consequently clinical development of BVM was suspended in 2010.

 Halted clinical development of BVM necessitates the need for a second-generation maturation inhibitor to overcome the problem of intrinsic drug resistance encountered by BVM. BVM targets an as yet undefined drug-binding pocket, which is hypothesized to be created upon higher-order multimerization of Gag during virus particle assembly. The significant technical challenge of obtaining high-resolution structural information of this hypothetical drug target makes rational structure-based drug design unfeasible at the current time. However, the need to develop improved maturation inhibitors has highlighted a need to further our understanding of the CA-SP1 region of Gag and its role in HIV-1 particle assembly. BVM, PF-46396, and their analogues can be utilized as tools to further explore drug-binding requirements to inform future strategies to improve drug resistance profiles. Development of BVM has provided evidence that small molecules to inhibit HIV-1 replication can target Gag cleavage sites. Four other cleavage sites are present in Gag and a genetic study predicted that a small molecule that blocks MA-CA cleavage maybe a particularly potent inhibitor of HIV-1 replication [119]. However, the intrinsic flexibility in Gag cleavage sites and wide variation in substrate sequence recognition by HIV PR may represent insurmountable problems for the future development of maturation inhibitors.

## Figures and Tables

**Table 1:** FDA approved protease inhibitors. Key protease resistance mutations sourced from the 2011 data review of HIV drug resistance by the international AIDS society USA [15].

Protease inhibitor	Year of FDA approval	Key resistance mutations
Saquinavir	1995	G48V, L90M
Ritonavir	1996	Used for boosting
Indinavir	1996	M46I/L, V82A/F/T, I84V
Nelfinavir	1997	D30N, L90M
Fosamprenavir	1999	I50V, I84V
Lopinavir	2000	V32I, I47V/A, L76V, V82A/F/T/S
Atazanavir	2003	I50L, I84V, N88S
Tipranavir	2005	I47V, Q58E, T74P, V82L/T, N83, I84V
Darunavir	2006	I47V, I50V, I54M/L, V76V, I84V
